# Novel Immune Microlens Imaging for Detection of Antigen and Antibody

**DOI:** 10.1155/2019/5474519

**Published:** 2019-04-18

**Authors:** Jiahui Liang, Xiaotian Ye, Jiang He, Jing Liu, Yaoxiong Huang, Feiyue Xing

**Affiliations:** ^1^Institute of Tissue Transplantation and Immunology, Department of Immunobiology, Jinan University, Guangzhou 510632, China; ^2^Department of Biomedical Engineering, Jinan University, Guangzhou 510632, China; ^3^School of Biomedical Engineering, Southern Medical University, Guangzhou 510515, China; ^4^School of Stomatology, Jinan University, Guangzhou 510632, China

## Abstract

Detection and analysis of antigen-antibody reaction is one of the most critical detection techniques in the fields of medicine, biology, environmental science, and food safety. Traditional and classical methods for detecting antigen and antibody encounter many problems, such as time-consuming, high cost, and low accuracy. A novel immune microsphere imaging technique by the microlens is used to test the changes of refractive index before and after antigen-antibody reaction. It can quickly perform qualitative and quantitative determination for antigen-antibody reaction without any labeling, premodification, postwashing, and expensive enzymes. Here, we feature and discuss its principle and advantages, structure of a microlens immunoassay instrument, and potential in measuring clinical samples. It is promising to be developed for application to diagnosis of clinical diseases.

## 1. Introduction

Common techniques available nowadays for antigen and antibody detection include enzyme-linked immunosorbent assay (ELISA), surface plasmon resonance (SPR), radioimmunoassay (RIA), colloidal gold immune chromatographic assay (GICT), indirect immunofluorescence assay (IFA), chemiluminescence immune assay (CLIA), and particle-enhanced turbidimetric immunoassay (PETIA). ELISA combines the amplification of the enzyme-catalyzed reaction and the specific reaction of the antigen and antibody with high accuracy and low cost, but its procedures are complicated with stringent condition control [1, 2]. SPR was developed in the 1990s to detect the interaction between biomolecules and other molecules, and it does not require any labeling and can get the result quickly, but its equipment is expensive and needs large sample volume [3]. RIA has the characteristics of high sensitivity, reliability, and low sample volume requirement. It is widely used in the detection of protein, enzyme, and other molecules, but the radionuclide is harmful to health and also alters biological activities of samples, leading to experimental error [4]. As a new type of immunoassay technique and one of the most common methods for detecting antigen and antibody, GICT is easy, simple, and quick with low cost, but its sample size is limited with low sensitivity [5–7]. CLIA, IFA, and PETIA are also widely used in the detection of antigen and antibody. Their common advantages are highly accurate, stable, easy, and quick, but with high cost and large sample volume [8–10].

An immune microlens imaging technique to test the change of refractive index can break the limitations of the above methods. It is rapid, sensitive, and simple with nonpollution and low cost for detecting and expected to be widely applied to primary medical institutions [11–14]. This technique consists of parallel light irradiation, high-resolution camera, intelligent analysis software, autofocus, and temperature control systems with a multiwell microlens sample test plate and can achieve multipass detection of antigen and antibody [12, 13]. The high-resolution camera system can meet the requirements of microlens imaging, and the use of temperature controller, autofocus, and automatic intelligent analysis systems can greatly reduce errors in experiments for accurate measurement.

## 2. Principle of Microlens Immunoassay

**Figure 1:**
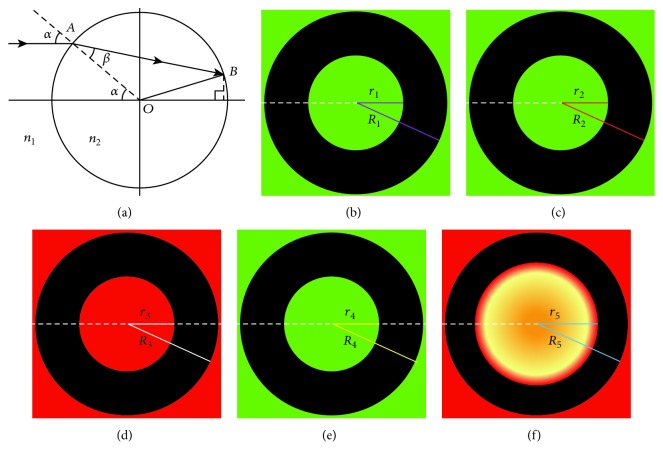
Immune microsphere imaging in various solutions by the microlens. (a) Basic principle of immune microsphere imaging. Images of a microlens are shown in water with the wavelength 532 nm at 25°C (b), in water with the wavelength 532 nm at 37°C (c), in water with the wavelength 633 nm at 25°C (d), in ethanol with the wavelength 532 nm at 25°C (e), or in serum with the wavelength 633 nm at 37°C (f), respectively.

Microlens is a cylindrical lens with one end of spherical surface and the other end is a plane surface. It has a strong amplification effect and significantly improves the imaging ability of traditional optical microscope [15–17]. When a microlens with a radius of *R* and refractive index (RI) of *n*_2_ is immersed in a solution of *n*_1_ (*n*_2_ > *n*_1_) and illuminated by plane light, due to the effect of refraction, its image is a round one with a dark ring at its edge [11, 12, 18]. The relationship between the radius *r* of the bright spot in the image and other parameters such as *R*, *n*_1_, *n*_2_, and the cylinder height *h* of the microlens is displayed as follows [11]:

(1) $$\begin{array}{c} r=R\sin\alpha -\left[R\cos\alpha +h\right]\frac{\sin\alpha \sqrt{1-k^{2}\sin^{2}\alpha }-k\sin\alpha \cos\alpha }{\cos\alpha \sqrt{1-k^{2}\sin^{2}\alpha +k\sin^{2}\alpha }}, \end{array}$$

where *α* is the incident angle of light to the spherical top of the microlens and *k* = *n*_1_/*n*_2__._ On the basis of this formula, the instantaneous changes of *n*_1_ of medium can be determined by measuring the radius *r* of the bright spot in the imaging and the radius *R* of the microlens. Since optical refraction takes place at the speed of light, any instant RI variation in the surrounding medium of a microlens can immediately induce a change in the radius *r* of the central bright spot. Therefore, the method can monitor instantaneous RI/concentration change with a high-speed camera for the imaging (Figure 1).

## 3. Structure and Functions of a Microlens Immunoassay Instrument

**Figure 2:**
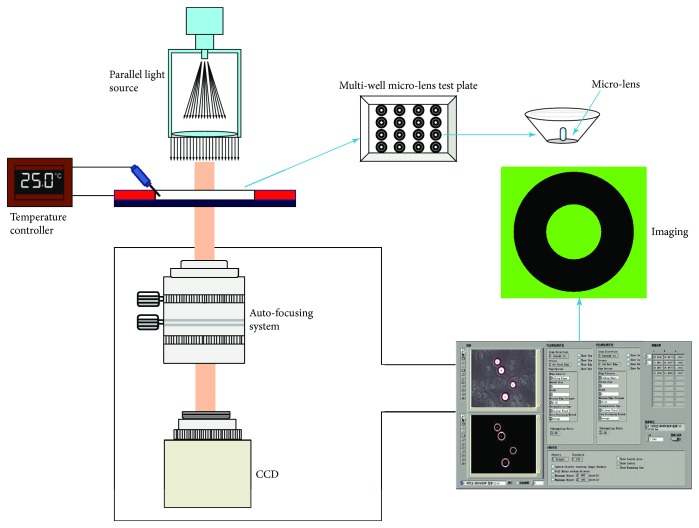
Structure of a microlens immunoassay instrument. It includes parallel light irradiation, autofocus, high-resolution imaging, intelligent analysis software, temperature control systems, a multi-well micro-lens test plate.

As shown in Figure 2, parallel light source, high-resolution imaging, automatic intelligent analysis, autofocus and temperature control systems, and a multiwell microlens test plate compose a microsphere immunoassay instrument [11]. When the parallel light source emits parallel light to the microlens, the high-resolution autofocus imaging system obtains an in-focus image in milliseconds. Then, the immune microsphere imaging is analyzed by the intelligent analysis software system to deduce values of *r* and *n*_1_ as well as concentration of antigen/antibody. Function of the temperature control system is to adjust different temperatures demanded for different antigen-antibody reactions. The whole process spends not more than 2 minutes.

### 3.1. Parallel Light Irradiation System

**Figure 3:**
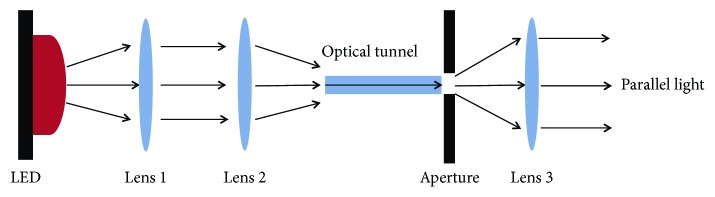
Principle of the parallel light irradiation system. The lens 1 and lens 2 focus the light emitted by LED. Then, the focused light goes through optical tunnel and aperture to be projected to the lens 3. Finally, the actual parallel light is acquired by lens.

LED as a source of parallel light produces a cylindrical parallel illumination area, which is similar to an original condenser with advantages of low cost, long life cycle, perfect luminous performance, and small scale [19]. As shown in Figure 3, actual parallel light can be received due to the refraction of lens [20, 21]. In the irradiation area of the LED, the lens can change direction and distribution of light by configuring relevant lens so that the actual parallel light is obtained (Figure 3).

### 3.2. High-Resolution Autofocus Imaging System

This system consists of a high-speed digital camera and an autofocus system. At present, the imaging speed of some commercial digital cameras has surpassed 10,000 frames per second [22]. Therefore, the high-resolution imaging system can achieve the real-time monitoring on dynamic changes of the RI in the solution. A high-pixel CCD camera plays an important role in obtaining a high-resolution microlens imaging [23, 24]. Cores of the autofocus system consist of image identification, image processing, and control parts [25]. The images collected by the camera system are analyzed, and the clarity evaluation is shown. According to this evaluation, the system is automatically driven to an appropriate area or direction until resolution of the acquired images satisfies the preset requirement.

### 3.3. Automatic Intelligent Image Recognition and Analysis System

The automatic intelligent analysis system mainly conducts image recognition and data analysis on the microlens imaging so as to monitor the instantaneous variation of *r* in its image and thereby deduce the refractive index change Δ*n* of the sample solution during the antigen-antibody reaction process. Its accuracy is closely related to the image quality, and parameters such as pixel, pixel size, and resolution directly affect the measurement of the RI [26]. If the CCD digital camera with ≥10 million pixel and small pixel size that reaches less than 3 nm and a microlens with radius of 600 *μ*m are exploited, refractive index change Δ*n* is determined by changes of the center bright spot ratio *r* and the ratio of the outer ring radius *R* so that the measured refractive index change Δ*n* can reach 10^−6^ [27].

### 3.4. Temperature Control System

The temperature control system is a transparent toughened glass board with a thin film of indium tin oxide and controlled by a high accuracy of a PID (proportional-integral-derivative) temperature controller. It enables the temperature of the sample in the multiwell microlens test plate to rapidly reach to a set value in 2 min and be maintained within change range of 0.1°C for making antigen-antibody reaction effective [28–30].

### 3.5. Microlens Test Plate

A multiwell microlens plate is specially designed for a microlens immunoassay instrument. It is made of polymethyl methacrylate (PMMA) material and is generally prepared as 2 or 16 trapezoidal wells to satisfy different detection requirements. Inside each well, there is a microlens with a radius of several hundred microns at its bottom. Application of the microlens plate provides an objective condition for multichannel detection. Since the diameter of the well underside is just about 2 mm, several microliters of sample solution are sufficient to drown the microlens for antigen-antibody detection.

## 4. Application of the Immune Microsphere Imaging System

### 4.1. Measurement of Antigen-Antibody Reaction

**Figure 4:**
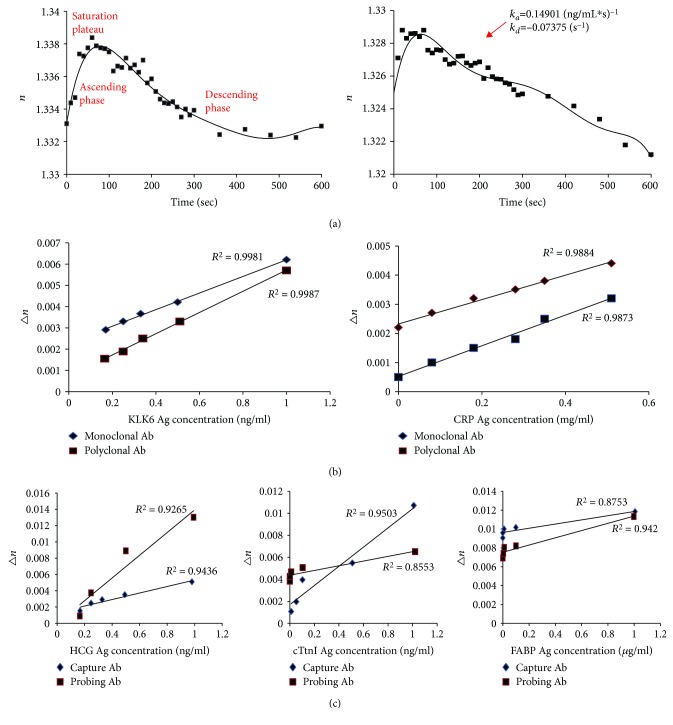
Refractive index changes with antigen-antibody reactions. (a) Relationships between the refractive index of IFN-*γ* or PAP antigen-antibody reaction and its time. (b) Relationships between Δ*n* and KLK6 or CRP antigen concentrations were determined, respectively, when its monoclonal Ab or polyclonal Ab was added to corresponding antigen. (c) Relationships between the Δ*n* and HCG and cTnI or FABP antigen concentrations were analyzed, respectively, when its capture Ab or probing Ab was added to corresponding antigen. Ag: antigen; Ab: antibody; Δ*n*: change of refractive index; IFN-*γ*: interferon-*γ*; PAP: placenta alkaline phosphatase; KLK6: kallikrein 6; HCG: human chorionic gonadotropin; cTnI: cardiac troponin; FABP: fatty acid binding proteins; CRP: C-reactive protein.

Huang and his colleagues [11] detected various types of antigen-antibody reactions and found out their regular features. First, refractive index changed with reaction time in the process of antigen (Ag)-antibody (Ab) reaction. Second, there were three phases in the variation of RI with reaction time, including rapidly increasing, relatively stable, and slowly declining periods. The first stage was related to combination of Ag and Ab so that the RI was increased suddenly with the Ag and Ab quick combination to form complexes. The maximum of RI is in the second phase. Third, concentration of the Ag or Ab had a great influence on the RI. Thus, by obtaining the RI change Δ*n* as a function of the Ag or Ab concentration, their contents in tested samples can be calculated by fitting curve. Fourth, different antibodies, such as capture Ab and probing Ab, also would influence the variation of RI. Using this technique to measure antigen-antibody reactions by several Ag-Ab systems, the relationships between the refractive index change Δ*n* and concentrations of several types of antigen-antibody solutions (interferon-*γ* (IFN-*γ*) Ag-Ab, placenta alkaline phosphatase (PAP) Ag-Ab, kallikrein 6 (KLK6) Ag-Ab, human chorionic gonadotropin (HCG) Ag-Ab, cardiac troponin (cTnI) Ag-Ab, fatty acid binding proteins (FABP) Ag-Ab, and C-reactive protein (CRP) Ag-Ab solutions) could be obtained (Figure 4). As we know that the association and dissociation of the antigen-antibody complex is a dynamic process, based on the curve of RI vs. time and calculation of the derivative of *n* with time, one can also obtain information about the association and dissociation rate constants *k*_*a*_ and *k*_*d*_ (Figure 4(a)) and other thermodynamic parameters by using the following equation:

(2) $$\begin{array}{c} \frac{dn}{dt}=k_{a}{Cn}_{\max}-\left(k_{a}C+k_{d}\right)n. \end{array}$$

### 4.2. Measurement of Clinical Samples

**Figure 5:**
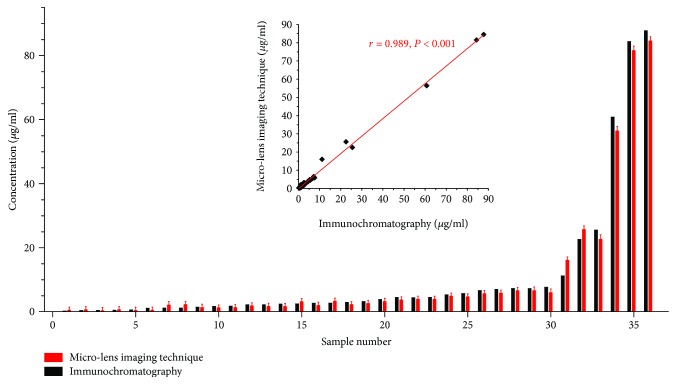
Measurement of 36 clinical samples for the C-reactive protein antigen using the immune microlens imaging in comparison with the immunochromatography.

The immune microsphere imaging technique was further utilized to test clinical samples. 36 clinical samples were detected by this one for concentrations of CRP Ag. Its relative standard deviation is about 2-10% in comparison with immunochromatography, and the correlation coefficient between the results acquired by the two ways reaches as high as 0.989 (Figure 5). It is well known that clinically hemolyzed serum samples can greatly impact accuracy of results detected by using traditional methods. On the contrary, images of the microlens in hemolyzed samples are still clear through this technique. The relative errors between the detected antigen values in the samples with hemolysis and the ones without hemolysis are just about 2%, indicating the less influence of the hemolyzed serum samples on the result accuracy [11].

## 5. Feasibility of the Immune Microsphere Imaging for Antigen and Antibody Detection

Most of antigens are proteins while a few are polysaccharides, nucleic acids, and other substances, and all of the antibodies are proteins. Since the protein contains a large amount of amido and carboxyl, these polar groups cause the colloidal particles to produce an electric charge due to the electrostatic action, and the particles with the same charge were repelled each other [31, 32]. At the same time, the polar groups that are strongly hydrophilic react with water molecules and form a hydration layer to produce a hydrophilic colloid, ensuring the protein does not agglomerate to form a precipitate so that the colloidal particles are uniformly dispersed in a solution [33–36].

When antigen is combined with antibody, the electric charge of colloidal particles reduces or disappears, and the hydration layer also disappears or becomes thin. The protein changes from the hydrophilic to hydrophobic colloid [37]. In the environment of electrolyte, colloidal particles further agglomerate to form the antigen-antibody complexes that can be realized by the eyes [38]. The refractive indexes of the hydrophilic and hydrophobic colloid are remarkably different. Therefore, this technique can monitor dynamic changes of the refractive index to judge whether there occur antigen and antibody reactions in a solution. A standard curve can be established according to a relationship between antigen and antibody concentration and their reaction time. The complex becomes larger with the process of antigen-antibody reaction, and the change of the refractive index also becomes more obvious. Using the standard curve, it is easy to quantify concentration of detected antibody or antigen.

It was demonstrated that an antibody that is complementary to epitopes of different antigens would indeed induce similar RI increment in Ag-Ab reaction with the microlens imaging immunoassay. As shown in Figures 4(b) and 4(c), antigen measurements were performed using capture Ab and probing Ab or monoclonal Ab and polyclonal Ab for a certain antigen. From the curves, we can see that there was no significant difference between the two kinds of antibodies in inducing *n* change during the Ag-Ab reaction, indicating that the microlens imaging immunoassay can use different kinds of antibodies, either capture or probing Ab and monoclonal or polyclonal Ab for detection. Nevertheless, monoclonal Ab is preferred for microlens imaging immunoassay, for it does not only induce greater *n* in reaction by its higher affinity to antigen but also reduce the possibility of cross-reaction false positive, so that antigens can be detected at lower concentrations [15]. Overall, cross-reaction is theoretically unavoidable for this technique like other means involved in immunoassay. In other words, specificity of the immune microlens imaging depends completely on that of selected antibodies against the target antigen. Therefore, it is very important to screen out appropriate antibodies and to optimize the Ag-Ab reaction system.

## 6. Conclusions

This immune microlens imaging technique can quickly and accurately measure the RI of different samples and monitor real-time changes of the RI in the process of antigen and antibody reaction. The RI alters with changes of the concentration of the Ag or Ab. Thus, according to the RI change Δ*n* as a function of the Ag or Ab concentration, the contents of samples can be calculated qualitatively and quantitatively without any labeling, premodification, postwashing, and expensive enzymes. Compared with conventional detection ways, the advantages of this method are accurate, reliable, quick (finished within several minutes), and simple without pollution. Besides, its detection limit is as low as pg/ml, which just requires several *μ*l enough to perform detection and it is also suitable for hemolyzed clinical samples that are difficult to be detected with traditional methods. What is more, it is nonintrusive to the samples. The parallel light irradiation system, high-resolution camera system, automatic intelligent analysis system, autofocus system, temperature control system, and microlens porous detection board compose the microsphere immunoassay instrument. It is small and easy to carry.

It is well known that the antigen-antibody reaction is greatly influenced by its own concentration, temperature, pH, and electrolyte solution. For this reason, these factors are taken into consideration in the microlens imaging system to keep data stable through balancing their changes. For example, this system can be optimized by selecting suitable materials for a test plate that offers an antigen-antibody reaction site and making the plate hydrophilic to avoid hydrophobic interference. The detecting accuracy can further be improved by adjusting antigen-antibody proportion, screening proper diluent, modifying them with metal ions, and so on.

Detection of antigen and antibody is relatively significant in the fields of biomedicine investigation, clinical diagnosis, drug analysis, food safety, and environmental monitoring. With people's concerns on environmental health, food safety, and healthcare, developing a sensitive instrument with low cost, high accuracy, small size, portability, and simple operation has become a pressing social need. Thus, this immune microlens imaging technique is promising to be broadly applied to various fields and appropriate to be especially popularized at community and countryside.
